# Bibliographical—Aide Memoirs of the Surgeon Dentist

**Published:** 1889-11

**Authors:** 


### EDITORIAL.

### Bibliographical.—Aide-Memoire of the Surgeon Dentist.

BY M. PAUL DUBOIS.

Director of L'Odontologie, President of the Society of Odontology of Paris, Professor of Special Therapeutics in the Dental School of Paris.

*Part First.—Therapeutics of Dental Caries. Second Edition, Paris, Bureaus de L'Odontolgie*, 1889.

The second edition under the above title of an exceedingly well arranged and practical book has come recently to hand. The author makes no claim to having brought forth a book of new ideas and methods, but a perusal of the work convinces one that it would lose much of its practical value if the gleaned material were left without a liberal intermingling of the author's ideas and directions for procedure. We translate the following from the preface:

"The substratum of this little book is taken from the course of Special Therapeutics, which has been, for the most part, under our charge during the years 1887 and 1888. The pathological part is a resume following the classical authors Magitot, Tomes, Wedl, Salter, Black, Miller. The therapeutical portion is the practice of the Dental School of Paris, as constituted by our masters M. M Levett and Poinsot and the group of young professors which the movement of the last few years has brought to light.

Some researches and personal ideas are added—explanation of the action of arsenious acid upon the inflamed pulp, and the modification of formulas employed up to the present time for its devitalization—a new mode of filling canals—the methodical employment of volatile and absorbent medicaments in teeth with decomposing pulps—the imperative necessity of a dressing which seals perfectly, if one wishes to apply the antiseptic method to the treatment of dental caries."

The author concludes his preface by stating that "There is no other manual in the French language condensing the principles of dental surgery with its latest transformations, consequently one may see reason for the existence of the Aide-Memoire under its new form."

The book begins by a description of office furniture, apparatus, and appliances which compose the usual modern outfit, a list of sixty four drugs and pharmaceutical preparations is given, and some excellent advice as to their care to avoid deterioration. All should be kept in small quantities, and many frequently renewed; others should be kept in colored bottles to prevent alteration by light, notably, iodine, iodoform chloroform, creosote, peroxide of hydrogen, essence of girofle, carbolic acid, and their compounds.

Caries is defined as "a special alteration of the tissue of the tooth, progressing from the periphery to the centre, occurring particularly upon teeth, or portions of teeth, abnormally constituted." Its etiology is classified into predisposing and determining causes. The predisposing causes are treated under four subdivisions, viz: 1st, Heredity; 2nd, Vices of development and of constitution of structure; 3rd, Insufficient alimentation or assimilation of phosphatic or calcific products, especially during the period of formation of the teeth; 4th, General debility, lymphatic condition, and repeated gestation. Determining causes under three subdivisions: 1st, Vital; 2nd, Chemical, and 3rd, Parasitic.

The degrees of decay are discussed under the following classification of the Paris School of Dentistry, "which is the same as the classification of Magitot, with one degree added, to distinguish between a diseased pulp and a pulp undergoing decomposition." "Four degrees. 1st degree, Alteration of the enamel; 2nd, Alteration passing through the enamel and attacking the dentine but leaving the pulp protected; 3rd, Perforating caries attacking the dentine as far as the pulp, which is either exposed or covered by softened dentine, and in a condition of lessened vitality; 4th, The pulp is destroyed and decomposed within its chamber as well as throughout the canals."

Considerable space is devoted to devitalized teeth and methods of their treatment. The author prefers arsenic acid to arsenious for devitalization, and does not favor the application of arsenic to the milk teeth under any circumstances. He considers immediate permanent root filling hazardous under almost any circumstances. His confidence in saving teeth which it has become necessary for the operator to devitalize, is set forth in the following paragraph, page 145: "Caries of the third degree, well treated, should not be liable to any recurrence of trouble, and the unsuccessful cases are, at the very most, two or three per thousand."

After stating his objections to fibres of cotton, silk, etc., as a means of carrying cement into root canals, the author describes his method of using aluminium wire and glycerized cement, which he describes as gliding without difficulty into the narrowest canals.

The work, as a whole, is closely and concisely written and is quite decided in its expression throughout, and one is not left in doubt as to what the author's views really are on any branch of his subject. We recommend a perusal of M. Dubois' book to any of our readers who may be familiar with the French language. R.

